# Metabolomic biomarkers for hepatocellular carcinoma: A systematic review

**DOI:** 10.1097/MD.0000000000028510

**Published:** 2022-01-21

**Authors:** Ningning Feng, Fatao Yu, Feng Yu, Yuling Feng, Xiaolin Zhu, Zhihui Xie, Yi Zhai

**Affiliations:** a Department of Infection Disease & Hepatology Ward, Zibo Central Hospital, Shandong, China; b Oncology Department, Zibo Central Hospital, Shandong, China.

**Keywords:** diagnosis and prognosis, HCC, metabolomics, systematic review

## Abstract

**Background:** Hepatocellular carcinoma (HCC) is a highly malignant cancer which lack of effective diagnosis and prognosis biomarkers, therefore surging studies focused on the metabolite candidates for HCC. The current study was designed to systematically review the metabolic studies for HCC, summarize the current available evidence and provide implication for further studies within this area. By systematically screening Pubmed and Embase, and eligibility assessment, we eventually included 55 pieces of studies. After summarized their characteristics, we reviewed them by 3 parts, regarding to the different biofluid they carried out the experiments. By collecting the candidates from all the included studies, we carried out pathway enrichment to see the representative of the reported candidates, as expected the pathway consistent with the current knowledge of HCC. Next, we conduct quality assessment on the included studies. Only 36% of the current evidence grouped as high quality, indicating the quality of metabolic studies needs further improvement.

## 1 Introduction

Hepatocellular carcinoma (HCC) as the most prevalent cancer ranks the fourth of the cancer-related mortality with 7,50,000 newly diagnosed patients per year.^[1]^ Unfortunately, 70% of them caught at late stage thus curative options are not available. Therefore, they could only have access to a palliative treatment. Sorafenib is currently the first-line targeted therapy for them which improved the overall survival of 10.7 vs 7.9 months comparing with placebo. The complexity of the disease is also represented by almost 160 driver genes were reported play a role in the progression of HCC.^[2]^ Considering the high mortality rate, insufficient diagnosis, as well as poor end stage intervention, the biomarker discovery field is rapidly expanding from previously proteomic study, metabonomic analysis to the recent deep sequencing strategies. However, a plenty of the candidates reported await further validation.

Metabolomics methodology is ideally designed to identify and estimate the changes endogenous metabolites exhaustively. With the rapid improvement of analytical technologies, it is convenient for researchers to screen the metabolome easily by tools such as mass spectrometry (MS) and proton nuclear magnetic resonance (^1^H NMR) to detect the biological changes during HCC. Recently, the metabolite changing has raised more and more attention. Intratumoral metabolites could not only potentiate the tumor progression, also could provide cross talk with cancer cell and the tumor microenvironment.^[3–5]^ For example, a recent study showed that tumor methionine metabolism could drive T-cell exhaustion in T cell, therefore inhibit the antitumor capabilities.^[6]^ Although a number of independent researchers have reported on metabolomics biomarkers of HCC,^[7–10]^ the insufficient cross-validation limits therefore clinical application.

The current study was carried out to systematically review the published data regarding to metabonomic changes of HCC patients. Not only re-evaluate those putative biomarkers proposed by academics, but also assess the quality of the previous metabonomic studies regarding to HCC. Secondly, as liver is the metabonomic hub of human, the regulation process of metabolome inside liver is crucial for uncovering the mechanism of HCC. Herein, we reported a systematic evaluation of previously reported candidate biomarkers for HCC, as well as assessed the quality of the relevant publications which could hopefully provide impact for subsequent metabonomic study.

## 2 Methods

### 2.1 Literature search strategy

Following the Preferred Reporting Items for Systematic Reviews and Meta-Analyses (PRISMA) and Meta-analysis of Observational Studies in Epidemiology (MOOSE) statements, publications relevant to this topic were initially searched from MEDLINE, EMBASE and Cochrane, by the end of May 2020. The following searching strategies were applied that terms related to “metabolomics” technique, “hepatocellular carcinoma”, adapting for individual database and their own search strategies.

### 2.2 Inclusion criteria and exclusion criteria

Articles fulfilled the following inclusion criteria were included:

1. participants had hepatocellular carcinoma with metabonomics were analyzed;
2. metabonomic techniques such as NMR or MS were adopted to construct metabolite profiles;
3. Only reported in English was included.

The following studies were excluded:

1. animal studies;
2. in vitro studies;
3. irrelevant to metabonomics;
4. not focusing on the metabonomic changes of HCC;
5. review articles.

### 2.3 Data extraction and analysis

Two authors (Ningning Feng, Fatao Yu) independently extracted the following information from the included studies: study profile (title, authors, year of publication, country), sample type (serum, plasma, urine, tissue), metabolomics strategies used, metabolites identified. Information regarding the patient characteristic which highly influenced either HCC or metabolites, such as the etiology, past medical history, HCC stage was also documented. An aggregate participant's data synthesis was performed with all included studies. Additionally, the metabolites were grouped and synthesized when more than 5 studies reported the whole complete metabolites profile, as most metabolite studies preferred to report statistically significant signals, rather than the overall metabolic data. Extracted data was synthesized to describe changes of metabolites in hepatocellular carcinoma patients. When data is eligible for synthesize to assess the changes of metabolites, the meta-analysis were performed via Revman 5.4.

### 2.4 Methodological quality assessment

Quality of each study was appraised independently by two authors based on the QUADOMICS for the evaluation of the methodological quality of studies on the diagnostic accuracy of “-omics”-based technologies 11, as well as the recent publication regarding to the quality of NMR 12. The detailed of the assessment criteria was shown in supplementary (See in Supplementary 1. Supplemental Digital Content, Quality assessment checklist). The quality of the study was defined as “high quality” once achieved 8/13, whereas those scored lower 8 were classified as “low quality”.

### 2.5 Ethical statement

This study is approved by Ethics Committee of Zibo Central Hospital.

## 3 Results

### 3.1 Characteristics of included studies

**Figure 1:**
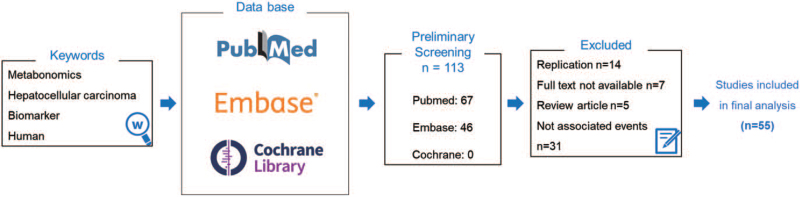
Flow chart of literature screening process.

A total of 113 hits initially reached from Pubmed and Embase. After removal duplications, title abstract screening, 58 articles were excluded. Finally, 55 studies entered in our systematic review. A diagram showing the detail literature evaluation procedure was shown in Figure 1. Due to the complexity of the metabolome research, the included studies are still of high diversity. Firstly, the metabolomic analysis often carried out on different types of biofluid, such as tumor tissue, urine, serum and plasma. Therefore, the sample collection and preparations were various among different matrix adopted. Additionally, study design, particularly the control group were distinctive. Most studies, observe the metabolomic characteristic of HCC, compared with healthy control, while other preferred the cirrhosis as a control. Also, some studies were designed to observe the relationship of metabonomic changes between HCC and the virus-induced hepatitis (particular HBV or HCV). Furtherly, the predominant metabolism analysis based on platforms, were widely adopted by the included studies, including GC-MS, NMR, LC-MS, UPLC-TOF-MS, etc. Therefore, we firstly gave a thoroughly summary on the characteristics of the included studies (Table 1).^[13–69]^ The following sub-group analysis was carried out based on the variables mentioned in the Table 1 which might cause the difference of metabolites among studies.

**Table 1:** Characteristics of included studies.

Biofluid	Platform	Control	Nationality	Ref.
Plasma	^1^H NMR	Health control; HCC; HCC-TACE; surgery	China	Chen et al 2016^[1]^
	MS	Heath control/Cirrhosis	Egypt	Nezami Ranjbar et al 2015^[2]^
			US	Di Poto et al 2017; Sanabria et al 2016^[3,4]^
			China	Lu et al 2019^[5]^
		Later recurrent vs early recurrent	China	Zhou et al 2014^[6]^
Tissue	MS	adjacent non-HCC area; adjacent cirrhosis	US	Ferrarini et al 2019^[7]^
			France	Teilhet et al 2017^[8]^
			China	Lu et al 2016; Huang et al 2013; Tang et al 2018^[9–11]^
Urine	MS	Heath control/Cirrhosis	China	Liang et al 2016; Shao et al 2014; Zhang et al 2013; Chen et al 2009; Wu et al 2009^[12–16]^
	^1^H NMR	Heath control/Cirrhosis	UK	Shariff et al 2016; Shariff et al 2011; Shariff et al 2010^[17–19]^
Serum	MS	Heath control/Cirrhosis	Korea	Kim et al 2019; Jee et al 2018^[20,21]^
			US	Barefoot et al 2019; Fitian et al 2014; Ressom et al 2012^[22–24]^
			Egypt	Xiao et al 2012^[25]^
			China	^[26–37]^
		iCCA, other gastric cancer	Spain	Banales et al 2019^[38]^
			France	Stepien et al 2016^[39]^
		HBV/HCV-cirrhosis or HCC	China	Gong et al 2017; Luo et al 2018; Wang et al 2014; Zhou, Wang, et al 2012^[40–43]^
			US	Bowers et al 2014; Baniasadi et al 2013^[44,45]^
			France	Liu et al 2018^[46]^
			Japan	Saito et al 2016^[47]^
	^1^H NMR	Early stage vs Advanced stage HCC	Italy	Casadei-Gardini et al 2020^[48]^
		Virus	France	Goossens et al 2016^[49]^
		Heath control/Cirrhosis	France	Fages et al 2015; Nahon et al 2012^[50,51]^
			US	Wei et al 2012^[52]^
Combination	MS	Heath control/Cirrhosis	China	Han et al 2020; Han et al 2019; Gao et al 2009^[53–55]^

### 3.2 Discriminate metabolites in plasma associated with HCC

There are 6 studies analyzed the metabolites via plasma, Chen^[30]^ and his colleagues developed their research to observe the altered metabolites which might be relevant to the recurrence or metastasis of HCC by comparing patients received clinical surgery or Transcatheter arterial chemoembolization (TACE). As a result, several metabolites associated with energy metabolism of amino acids (AA), glucose, and lipids were reported. It seems that metabolites could be a promising biomarker to predict the prognosis of HCC, however more evidence is needed considering it is the only 1 included study reported the metabolites change of surgery process on the NMR spectra. On the other hand, 6 pieces of work analyzed the metabolic profile in the MS setting. Five studies reported the metabolomic changes of HCC compared with health control or cirrhosis patients. After combination, there are 24 metabolites are upregulated in HCC group comparing to the cirrhosis controls, while 11 metabolites showed significantly downregulation in HCC patients. The reported pathway analysis indicated that biological process behind the changes of metabolites are energy metabolism like glycolysis, biosynthesis of amino acids, pyruvate, and degradation of glutamine, glutamate, as well as the TCA cycle. Since only 4 studies compared the difference between HCC group and their cirrhosis control group, and not all the studies provide the detail value of metabolic profile, we did not synthesis the data to observe the exact efficacy of these markers.

### 3.3 Metabolism observation via tumor tissue

Not too many studies carried out on the metabolism difference between tumor tissue, and adjacent non-tumor area. In our study, only 5 studies reported the metabolic characteristics of tumor itself all in a MS platform. As the complexity of the disease, the de novo metabolism profile is also complex. Included studies reported plenty metabolic changes inside the liver. Particularly, Huang and his colleagues collected 50 pairs of liver cancer samples and the matched normal tissue.^[51]^ After metabolomic discovery, they identified 103 metabolites, ranging from conventional fatty acids, amino acids, bile acids, to other compounds, such as carbohydrates, carnitines and others which cannot be grouped. The pathway enrichment also implied that several metabolic pathways were modified by the tumor cell. Such as metabolism of glycolysis, TCA, amino acids glutamine, glutathione, branched-chain amino acids (BCAA), the aromatic amino acids (ArAA) were changed accordingly.

### 3.4 Different metabolism expression in urine associated with HCC

In general, 8 studies collected the urine sample from their cohort to conduct the metabolic exploration. While 3 studies used ^1^H NMR platform for metabolites discovery, it seems the 3 studies from the same research group in United Kingdom.^[36,62,64]^ There are also 5 studies adopted MS spectra for metabolite profile identification. After further combination, there are 38 metabolites reported which showed increase in the HCC group, whereas 14 metabolites from urine sample downregulated in HCC group compared with the cirrhosis counterpart. Pathway enrichment from those 5 studies, suggest that the most influenced biological process of HCC is related to bile acid biosynthesis, bile secretion, bile acid metabolism, and glucose-alanine cycle. However, not all the included studies focusing on urine metabolism profile provide the whole picture of metabolism changing, some of them only claimed the observed the changings in metabolites without showing the quantification results, which made the data synthesis impossible for these research.

### 3.5 Characteristics of serum metabolism profile associated with HCC

Studies conducted on serum metabolism are the most predominant in this area. Five studies were designed via NMR platform, however their distinctive cohort setting make it impossible to synthesis their metabolism profile. Briefly, Casadei group interested in the progression of the disease, therefore they compared the different metabolites between early-stage HCC and advanced stage.^[13]^ While Goossens cared more about the difference between virus-related hepatitis and HCC, therefore their comparison carried out among these cohorts.^[23]^ Also, others used health control or chronic cirrhosis as the control.

**Figure 2:**
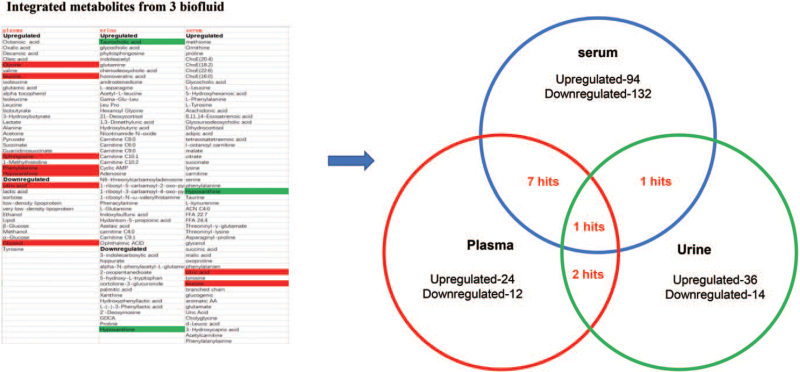
Integrated metabolites from included studies.

**Figure 3:**
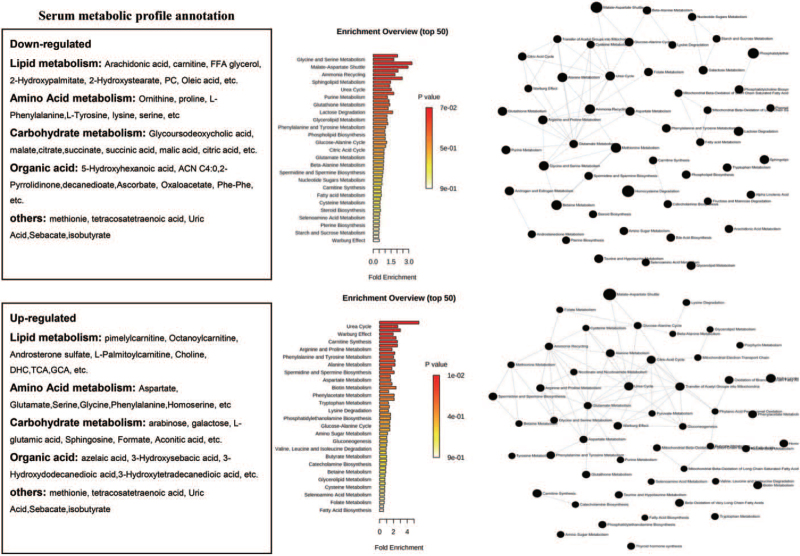
Serum metabolic profile annotation.

Apart from the NMR experiments, majority of the included studies (n = 28) preferred MS to perform the metabolome discovery. After systematically reviewed these publications, a total of 92 upregulated metabolites and 128 downregulated metabolites were retrieved, representatives are summarized in Figure 2. The involved metabolites ranging from carbohydrate metabolome, amino acid metabolism, lipid metabolism, as well as organic acid metabolism (Fig. 3). To further summarize the evidence on discriminated metabolites associated with HCC, we tried to carry out the meta-analysis on the studies provide statistical analysis regarding to the metabolite concentration. However, these reported changing metabolites are various, cannot fulfill the requirement for meta-analysis.

Therefore, the integrative analysis was adopted. We systematically summarized the reported metabolome changes by serum studies. Divide the regulated markers by their functional category, such as amino acid metabolism, lipid metabolism, carbohydrate metabolism, organic acid, and others. We carried out the pathway annotation by MetaboAnalyst (https://www.metaboanalyst.ca/MetaboAnalyst/home.xhtml). As the Figure 3 showed that the down regulated metabolites highly influenced most metabolism processes, such as amino acid, glucose, fatty acids, as well as their synthesis was influenced. Compared with the decreased metabolites, the increasing markers in HCC also indicated the tumor metabolism dysfunction effectively. For example, after the enrichment the significantly changed metabolites are mostly involved in urea cycle, Warburg effect, consistent with our conventional knowledge about HCC. During the disease progression, the urea cycle is highly injured. Also, the Warburg effect is the predominant characteristic, particularly for solid tumor such as HCC.

### 3.6 Quality assessment on the included studies

**Figure 4:**
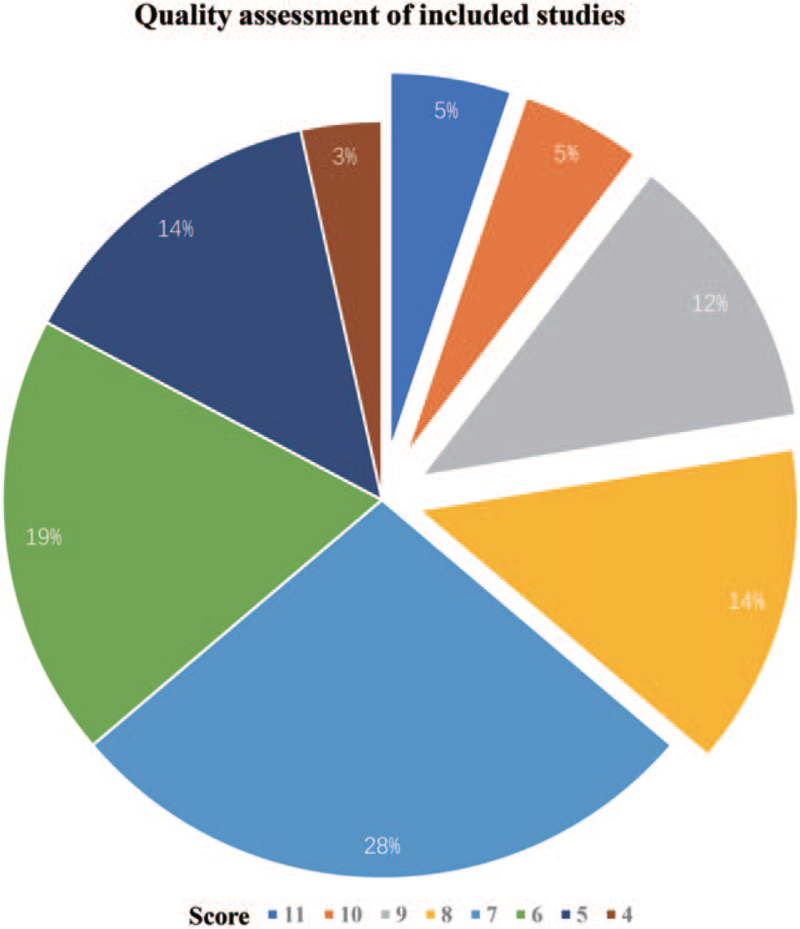
Quality assessment for the included studies.

Considering the failure on meta-analysis, we next focusing on the quality related to metabolome studies in this area. We totally included 58 studies, which is, to some extent, over-elaborate. However, limited number of the studies provides a comprehensive information for us to synthesis their results. Considering the specificity of metabolomic study, we firstly employed the QUADOMICS, combined with the quality assessment recommended by STROBE statement. We thoroughly assessed all the studies documented all the shortcomings, hope those assessment could provide further implication for the future metabonomic studies. Problems regarding to the included studies were shown in Table 2. As the quality score in Figure 4 showed that only 36% of the included publications showed high quality with a quality score over 8, while the others grouped to a low quality, indicated that most of the studies still need improvements.

**Table 2:** Common problems for the included studies.

Category	Problem
Selection	-No detailed inclusion criteria and exclusion criteria was shown;-The principle for control selection (age, gender)
Patient information	-Disease history-HCC stage-medication might influence the metabolite
Sample collection and preparation	-The time period for collecting sample;-The storage time, procedure, between inpatient and outpatient, particularly the health control;-Before analysis, how to prepare the sample, refreezing cycle, timing and sorting of speeding time.
Cross-validation	It is recommended to enroll a new cohort patient to validate the previous observation.
Reporting	It is highly recommended for the authors to report the experimental setting, as well as the statistical analysis data along with the metabolites.

## 4 Discussion

It is widely accepted that the liver plays a critical role in our metabolism. Therefore, the metabolome study focusing on liver could provide us a big landscape for the whole metabolomic activity inside our body. Therefore, plenty of researchers carried out metabolism investigation in liver. As the most predominant end-stage liver disease, HCC ranked as one of the most leading causes of cancer death. Because of the limited diagnosis strategy, most patients diagnosed HCC initially at the late stage; therefore they lost best chance for surgery, which on the other hand, aggravate the mortality of HCC. So far, the most valuable biomarker for this disease is alpha-fetoprotein (AFP). However, it lacks sensitivity and cannot monitor the disease progression either. Effective biomarkers for HCC are needed for many years. Recently, a great many academics have been keeping seeking the ideal biomarker from metabolome, publications are surging. With this background, we set up this systematic review. The objective of our study was to summarize the current knowledge within metabolomic study in HCC. At the same time, we also want to see if there is any ideal biomarker candidate which has already been discovered and needs further popularization.

By 2015, Dr Eholmes has already reviewed the promising markers for HCC from a proteomic and metabonomic view.^[70]^ As 5 years had passed, the technologies improved a lot; therefore we conducted this investigation to see if we can have more information particularly on metabonomic discovery. At the same time, we developed this study due to the quality of metabolomic study has recently been discussed by academics,^[12]^ particularly, for the reporting of statistical data for NMR analysis. We wondered if the mounting evidence in HCC, also encountered this difficult, if the current available data enough to provide us new insights for metabolites profile of HCC.

As the results showed, during the past 10 years, 55 publications carried out to observe the metabolic change in HCC. The investigation mainly focused on serum, plasma, and urine. With these 3 to 5 years, due to the multi-omics has been put forward; the metabolome of de novo HCC has also been investigated. However, due to the specificity of tumor tissue, the metabolome studies from tumor cannot be combined with previous biofluid discovery. Therefore, we did not combine these results with those biofluid based study. After combined the different metabolites by urine, plasma and serum, we found there is only one shared hit of the three parts. Indicating the metabolomics from these biofluids is various. However, this discrepancy might also due to the amount of evidence. Our summaries for the included studies showed that large numbers of studies focusing on serum, so left only limited parts for plasma and urine. Furtherly, it is hard to synthesis all the results, since the analysis platforms are different, as well as the study designs are different case by case. Apart from these, several factors could also influence the outcome of the study. Firstly, from the methodology aspects, the machines used for metabolites analysis, such as LC–MS, GC–MS, or targeted MS analysis could influence the results a lot. Secondly, other information like the data acquisition process, the method used for metabolites screening could cause those difference. Thirdly, metabolites might also exert difference since different objectives of each study bearing regional and diet characteristic.

We tried to deeply investigate the studies regarding to serum. After the systematically review, 92 upregulated metabolites and 128 downregulated metabolites were retrieved, respectively. However, among all the included studies about serum, only seven reported a statistical changing of metabolites. Others only showed the expression trend of the candidates, which makes the reassessment impossible. Therefore, it is impossible to conduct meta-analysis with the available data. Next, by using the different expression profile, we did the metabolome analyze to see if the current expression difference might consistent with the pathology of HCC. As the pathway enrichment indicated, the HCC group showed a decreased metabolism of glucose, fatty acid, as well as key glucose, as well as their biosynthesis. On the other hands, pathway related to urea cycle, Warburg effect were effectively enhanced.

These results consistent with our current knowledge to this disease, which indicated that metabolome can be a promising approach to predict the incidence and prognosis of HCC. In the future study of HCC patients, researchers should also focus on the metabolites changing, as it may also indicate the severe changes inside. With the discover of enhanced pathway, it may provide new targets for the treat of this disease.

Additionally, it is an indispensable part for systematic review to do the quality assessment. As our results showed, over 60% of the included studies grouped as low quality. This particular result reminded us it is important before the metabolomic studies, the experiments should be well-designed, especially for those confounders which heavily influenced the metabolites. As long as the sample collection process, the detail should document clear, as metabolites are really unstable, which highly influenced the repeatability of the study. 5 years ago, Dr Eholmes highlighted that it is important to carry out the validation study along with the metabolite discovery.^[70]^ However, 5 years later, still a majority studies reported their studies without further validation. Furtherly, the metabolomic data particularly with statistical analysis are highly recommended for sharing after publication, as it could provide more impact for future review.

There are limitations of the current study, as we have discussed in the earlier version, 60% of the included study assessed as low quality, which makes the conclusion of these studies are questionable. Additionally, as the methodology of metabolites analysis is varied among studies, thus the combined results are suspicious. Moreover, since the metabolites can be influenced by a number of characteristics, such as gender, region, diet, etc. Thus, the metabolites should be screened based all the clinical information has been balanced.

## 5 Conclusion

In summary, in the current study, we systematically reviewed current evidence regarding to metabolomic changes associated with HCC. A large number of studies has been published within this scope, with a great number of metabolic candidates has been proposed as a promising biomarker for HCC. However, none of them has been popularized to the clinics, indicating the evidence is weak. By systematically reviewed that evidence; the quality of those metabolic studies is highly needed for improvement.

## Author contributions

**Conceptualization:** Yi Zhai.

**Data curation:** Xiaolin Zhu.

**Formal analysis:** Ningning Feng, Yuling Feng.

**Funding acquisition:** Yi Zhai.

**Investigation:** Feng Yu.

**Project administration:** Fatao Yu, Feng Yu.

**Software:** Fatao Yu, Zhihui Xie.

**Writing – original draft:** Ningning Feng.

## Supplementary Material

Supplemental Digital Content
