# Supplementary material for: Metabolomic biomarkers for hepatocellular carcinoma: A systematic review
Source: Medicine (Baltimore). 2022 Jan 21;101(3):e28510. doi: 10.1097/MD.0000000000028510 (PMC8772637; doi:10.1097/MD.0000000000028510)
Supplement: Supplemental Digital Content [file medi-101-e28510-s001.docx]

| **Item** | **instruction** | **score** |
| --- | --- | --- |
| Selection criteria | Describing the detail information about exclude and include | Yes=1  No=0  Unclear=0 |
| Socio demographic | Sex, ethnicity, etiology, disease stage, medical history | 1/item (n=5) |
| Type of sample | Serum, plasma, urine, tissue | Yes=1  No=0  Unclear=0 |
| Procedure of sample, timing of sample |  | Yes=1  No=0  Unclear=0 |
| Handling and pre-analytical procedure reported in sufficient detail |  | Yes=1  No=0  Unclear=0 |
| Metabolomic techniques | numerical result formats;  full metabolites profile included;  statistical set | 1/item (n=3) |
| Cross-validation |  | Yes=1  No=0  Unclear=0 |

**Supplementary 1. Quality assessment checklist**
